# Предикторы выживаемости пациентов с АКТГ-эктопированным синдромом

**DOI:** 10.14341/probl13144

**Published:** 2022-12-20

**Authors:** О. О. Голоунина, Ж. Е. Белая, Л. Я. Рожинская, М. Ю. Пикунов, А. А. Маркович, Л. К. Дзеранова, Е. И. Марова, Н. С. Кузнецов, В. В. Фадеев, Г. А. Мельниченко, И. И. Дедов

**Affiliations:** ФГАОУ ВО «Первый Московский государственный медицинский университет им. И.М. Сеченова» Минздрава России (Сеченовский Университет); ФГБУ «Национальный медицинский исследовательский центр эндокринологии» Минздрава России; ФГБУ «Национальный медицинский исследовательский центр эндокринологии» Минздрава России; ФГБУ «Национальный медицинский исследовательский центр хирургии им. А.В. Вишневского» Минздрава России; ФГБУ «Национальный медицинский исследовательский центр онкологии им. Н.Н. Блохина» Минздрава России; ФГБУ «Национальный медицинский исследовательский центр эндокринологии» Минздрава России; ФГБУ «Национальный медицинский исследовательский центр эндокринологии» Минздрава России; ФГБУ «Национальный медицинский исследовательский центр эндокринологии» Минздрава России; ФГАОУ ВО «Первый Московский государственный медицинский университет им. И.М. Сеченова» Минздрава России (Сеченовский Университет); ФГБУ «Национальный медицинский исследовательский центр эндокринологии» Минздрава России; ФГБУ «Национальный медицинский исследовательский центр эндокринологии» Минздрава России

**Keywords:** АКТГ-эктопированный синдром, нейроэндокринная опухоль (НЭО), гиперкортицизм, предикторы, летальность, выживаемость

## Abstract

ЦЕЛЬ. Определить прогностически значимые факторы, влияющие на выживаемость больных с АКТГ-эктопированным синдромом.МАТЕРИАЛЫ И МЕТОДЫ. Многоцентровое ретроспективное исследование когорты пациентов с АКТГ-эктопированным синдромом. Конечной точкой исследования стал летальный исход пациента от любых причин. С целью выявления предикторов развития неблагоприятного исхода проведен однофакторный и многофакторный регрессионный анализ. Для определения прогностических пороговых значений отдельных предикторов использовался ROC-анализ. Анализ выживаемости проводили по методу Каплана–Майера. Статистическая обработка данных осуществлялась в IBM SPSS Statistics 23.РЕЗУЛЬТАТЫ. В исследование включен 151 пациент (92 женщины, 59 мужчин) в возрасте на момент диагностики заболевания от 12 до 76 лет (Ме 40 лет [28; 54]). Медиана периода наблюдения — 50 мес [13; 91], максимально — 382 мес. В 92 случаях (60,9%) первичный очаг локализовался в легком, в 17 (11,3%) — в средостении, в 8 — в поджелудочной железе, в 5 — в надпочечнике, по 1 случаю — в слепой кишке и червеобразном отростке, 1 — медуллярный рак щитовидной железы, 26 больных (17,2%) — с опухолью неустановленной локализации. Первичный очаг удален у 101 пациента (66,9%). Двусторонняя адреналэктомия по жизненным показаниям выполнена 42 (27,8%) пациентам. Метастазы выявлены в 23,2% случаев (n=35). Рецидив заболевания наблюдался в 24,4%, долгосрочная ремиссия сохранилась у 64 пациентов (74,4%). Летальные исходы наступили у 42 (28%) пациентов в возрасте от 27 до 81 года. Средний возраст выживших составил 47,0±15,2 против 53,5±15,6 года у умерших (р=0,022). Время до наступления летального исхода с момента постановки диагноза в среднем 32 мес, Ме 16,5 мес [7; 54]. Непосредственное влияние на выживаемость больных оказывают: возраст установки диагноза ≥51 года (OR 4,493; 95% ДИ 2,056–9,818, р<0,001), локализация нейроэндокринной опухоли (НЭО) в легком (OR 0,281; 95% ДИ 0,119–0,665; р=0,004), наличие метастазов (OR 2,489; 95% ДИ 1,141–5,427; р=0,022), концентрация свободного кортизола в вечерней слюне ≥122,2 нмоль/л (OR 2,493; 95% ДИ 1,014–6,128; р=0,047).ЗАКЛЮЧЕНИЕ. Наиболее частым источником эктопической продукции АКТГ являются НЭО бронхолегочной локализации, демонстрируя наилучшие результаты хирургического лечения и показатели выживаемости. На прогноз пациентов с АКТГ-эктопированным синдромом оказывают влияние возраст установки диагноза, локализация НЭО, наличие метастазов и концентрация свободного кортизола в вечерней слюне.

## Abstract (en)

AIM: To determine significant factors affecting the survival of patients with ectopic ACTH syndrome (EAS).MATERIALS AND METHODS: A multi-center, observational study with a retrospective analysis of patients with EAS. The end point of the study was the fatal outcome of patients from various causes. In order to identify predictors of survival or mortality, univariate and multifactorial Cox regression analyses were carried out. ROC-analysis was used to determine the prognostic threshold values of individual predictors. The survival analysis was carried out using the Kaplan-Mayer method. Statistical data processing was carried out by using IBM SPSS Statistics 23.RESULTS: The age of patients at the time of diagnosis ranged from 12 to 76 years (Me 40 years [28;54]). The age of the studied population was 55 years [38; 64] for women and 42 years [32; 54] for men. The median period of observation was 50 months [13;91], with a maximum follow-up of 382 months. 92 patients (60,9%) had bronchopulmonary NET, 17 (11,3%) — thymic carcinoid, 8 — pancreatic NET, 5 –pheochromocytoma, 1– cecum NET, 1– appendix carcinoid tumor, 1 — medullary thyroid cancer and 26 (17,2%) patients had an occult NET. The primary tumor was removed in 101 patients (66,9%). Bilateral adrenalectomy was performed in 42 (27,8%) cases. Metastases were revealed in 23,2% (n=35) of patients. Relapse of the disease was observed in 24,4%, long-term remission was preserved in 64 patients (74,4%). Death occurred in 42 patients (28%). The average age of survivors was 47,0±15,2 versus 53,5±15,6 years for the deceased (p=0,022). The average survival time from diagnosis for the deceased was 32 months, Me 16,5 months [7;54]. Multivariate analysis revealed that the following factors have a direct impact on survival: age of diagnosis ≥51 years (OR 4,493; 95% CI 2,056–9,818, p<0,001), bronchopulmonary neuroendocrine tumor (NET) (OR 0,281; 95% CI 0,119–0,665, p=0,004), the presence of distant metastases (OR 2,489; 95% CI 1,141–5,427, p=0,022), late-night salivary cortisol (LNSC) ≥122,2 nmol/L (OR 2,493; 95% CI 1,014–6,128, p=0,047).CONCLUSION: The prognosis of patients with EAS is influenced by the age of diagnosis, NET localization, distant metastases and level of LNSC. The most common cause of ectopic ACTH syndrome was bronchopulmonary NET which was associated with the best survival rate.

## ОБОСНОВАНИЕ

АКТГ-эктопированный синдром — крайне редкое и одно из наиболее тяжелых эндокринных заболеваний, обусловленное избыточной продукцией адренокортикотропного гормона (АКТГ) и, значительно реже, кортикотропин-рилизинг-гормона нейроэндокринной опухолью (НЭО), что формирует клиническую картину эндогенного гиперкортицизма. Заболевание составляет 5–20% всех случаев АКТГ-зависимого гиперкортицизма, с частотой примерно 0,2–1,0 на 1 млн населения в год [1][2].

Своевременная постановка диагноза, выбор наиболее оптимальной тактики лечения в зависимости от локализации патологической гиперпродукции АКТГ, коррекция осложнений гиперкортицизма являются ключевыми задачами ведения пациентов сданным эндокринным заболеванием, во многом определяя прогноз и качество жизни. В последние годы по мере усовершенствования методов топической диагностики процент оккультных опухолей имеет тенденцию к снижению, однако, несмотря на неоднократно проведенные обследования, примерно у 10–30% пациентов источник АКТГ-эктопированного синдрома остается неустановленным [3][4].

В опубликованном ранее исследовании самой большой серии наблюдений пациентов с синдромом эктопической продукции АКТГ проведен детальный анализ клинического течения заболевания, а также проанализированы ближайшие и отдаленные результаты хирургического лечения [4], однако в настоящее время в литературе недостаточно данных, оценивающих вклад различных факторов в определение прогноза заболевания и риска развития неблагоприятных событий у больных с АКТГ-продуцирующими НЭО различной локализации.

## ЦЕЛЬ ИССЛЕДОВАНИЯ

Определить прогностически значимые факторы, влияющие на выживаемость больных с АКТГ-эктопированным синдромом; провести анализ результатов хирургического лечения и длительного динамического наблюдения когорты пациентов.

## МАТЕРИАЛЫ И МЕТОДЫ

## Место и время проведения исследования

Место проведения. Исследование проведено на базе отделения нейроэндокринологии и остеопатий ФГБУ «Национальный медицинский исследовательский центр (НМИЦ) эндокринологии» Минздрава России.

Продолжительность исследования. Ретроспективный набор материала осуществлялся с октября 2020 г. по май 2022 г. Глубина поиска составила 30 лет.

## Изучаемые популяции

В исследование включены пациенты с установленным клиническим диагнозом АКТГ-эктопированного синдрома (МКБ-10: Е24.3) в период с 1990 по май 2022 гг.

Критерии включения: мужской и женский пол, АКТГ-зависимый эндогенный гиперкортицизм, подтвержденный в соответствии с федеральными клиническими рекомендациями [5] как минимум двумя лабораторными тестами: превышение верхней границы референсного интервала для свободного кортизола в суточной моче и/или кортизола в образце слюны, собранной в 23:00, или кортизола крови, взятого в 23:00, и/или отрицательная малая проба с 1 мг дексаметазона (отрезная точка 50 нмоль/л); концентрация АКТГ в утренние часы ≥10 пг/мл; отрицательный результат селективного забора крови из нижних каменистых синусов с использованием стимуляционного агента (градиент АКТГ центр/периферия <2 до стимуляции и <3 после стимуляции десмопрессином), и/или отрицательный результат большой дексаметазоновой пробы (снижение уровня кортизола крови менее чем на 60% исходного после приема накануне 8 мг дексаметазона).

Критерии исключения: другие верифицированные формы гиперкортицизма.

## Способ формирования выборки из изучаемой популяции

Способ формирования выборки — сплошной.

## Дизайн исследования

Многоцентровое исследование с ретроспективным анализом данных.

Конечной точкой в разделе исследования, посвященном изучению предикторов выживаемости, считали летальный исход пациента от любых причин.

## Описание медицинского вмешательства

Пациентам с предварительно установленной локализацией НЭО выполнялось хирургическое лечение: операции на легких (лобэктомия, сегментэктомия) проводились в отделении торакальной хирургии НМИЦ хирургии им. А.В. Вишневского, в Национальном медико-хирургическом центре им. Н.И. Пирогова или в Научно-исследовательском институте скорой помощи им. Н.В. Склифосовского, удаление опухоли переднего средостения — в отделении эндокринной хирургии НМИЦ эндокринологии, в НМИЦ онкологии им. Н.Н. Блохина или в Московском научно-исследовательском онкологическом институте им. П.А. Герцена, удаление опухоли надпочечника и гемиколэктомия по поводу новообразования слепой кишки — в НМИЦ эндокринологии, удаление образования поджелудочной железы — в НМИЦ хирургии им. А.В. Вишневского, тиреоидэктомия — в НМИЦ онкологии им. Н.Н. Блохина.

В послеоперационном периоде взятие проб выполнялось на 1-е сутки, при нормализации показателей — через сутки вплоть до выписки пациента из стационара. При снижении показателей ниже референсных значений назначалась заместительная терапия глюкокортикоидными гормонами, в случае выполнения двусторонней адреналэктомии — дополнительно заместительная терапия минералокортикоидными гормонами.

Восьмидесяти пяти пациентам, включенным в исследование, для дифференциальной диагностики между болезнью Иценко–Кушинга и АКТГ-эктопированным синдромом выполнялся селективный забор крови из нижних каменистых синусов с использованием стимуляционного агента (Desmopressin Acetate 4 мкг в 1 мл) в дозе 8 мкг. Забор венозной крови из правого, левого нижних каменистых синусов и нижней полой вены проводился одномоментно с экспозицией в 5 мин до введения десмопрессина в/в и 3 раза с интервалом в 3, 5 и 10 мин после введения десмопрессина в дозе 8 мкг [6]. В указанных интервалах времени кровь исследовалась на АКТГ. Лабораторная оценка концентрации уровня пролактина и АКТГ в венозной кровииз нижних каменистых и кавернозных синусов относительно концентрации этих гормонов в периферической пробе использовалась для контроля положения катетера. Градиент пролактина ≥1,5 свидетельствовал об адекватной установке катетера [6]. Градиент АКТГ центр/периферия ≤2 до стимуляции и ≤3 после стимуляции десмопрессином свидетельствовал в пользу АКТГ-эктопированного синдрома [7].

## Методы

Гормональное исследование АКТГ (референсный интервал: утро 7,2–63,3 пг/мл, вечер 2–25,5 пг/мл), кортизола в сыворотке крови в 23:00 (64–327 нмоль/л), определение свободного кортизола в вечерней слюне (0,5–9,6 нмоль/л) проводилось электрохемилюминесцентным методом на анализаторе Cobas 6000 Module e601 (Roche); измерение свободного кортизола в суточной моче (100–379 нмоль/сут) — иммунохемилюминесцентным методом на аппарате Vitros ECi.

Поиск новообразования, секретирующего АКТГ, включал проведение мультиспиральной компьютерной томографии, соматостатин-рецепторную сцинтиграфию и однофотонную эмиссионную компьютерную томографию, совмещенную скомпьютерной томографией в режиме «все тело» с ¹¹¹In-октреотидом, 123I-метайодбензилгуанидином или 99mTc-тектротидом и/или совмещенную позитронно-эмиссионную и компьютерную томографию с DOTA-конъюгированным радиофармпрепаратом (68Ga-DOTA-TATE). Диагноз верифицировался результатами патоморфологических исследований (гистологическое и иммуногистохимическое исследования операционного материала). Гистопатологический диагноз для каждой локализации первичной опухоли устанавливался в соответствии с критериями классификации НЭО Всемирной организации здравоохранения (ВОЗ). Степень злокачественности НЭО определяли в соответствии с критериями ВОЗ на основании величины индекса пролиферативной активности опухолевых клеток (индекс Ki67) и митотического индекса в 10 репрезентативных полях зрения (2 мм2). Во всех случаях эктопическая продукция АКТГ опухолями подтверждалась иммуногистохимическим методом по экспрессии этого гормона в клетках удаленной опухоли [8].

## Критерии оценки исходов

Все пациенты находятся на регулярном учете, что позволяет динамически контролировать их состояние. Факт летального исхода регистрировался по наличию данных медицинской документации, непосредственного контакта с родственниками пациента.

Критериями ремиссии АКТГ-эктопированного синдрома являлись: лабораторно подтвержденная надпочечниковая недостаточность (снижение кортизола крови утром ниже референсных значений) или нормализация уровня кортизола крови в раннем послеоперационном периоде, восстановление ритмов АКТГ и кортизола, нормализация уровней кортизола в суточной моче и/или слюне вечером, регресс клинических проявлений эндогенного гиперкортицизма в позднем послеоперационном периоде; отсутствие вторичных опухолевых очагов.

Отсутствие ремиссии заболевания, так же, как и рецидив АКТГ-эктопированного синдрома, устанавливались при наличии любых двух критериев: повышение уровня свободного кортизола в слюне, собранной в 23:00, повышение концентрации свободного кортизола в суточной моче, отрицательная малая проба с 1 мг дексаметазона в сочетании или без с возвратом клинической картины гиперкортицизма или отсутствие регресса клинических проявлений эндогенного гиперкортицизма в случае исключения передозировки или введения экзогенных глюкокортикоидов; продолженный рост опухоли или признаки наличия опухолевой ткани по результатам инструментальных исследований и радионуклидных методов диагностики; появление вторичных очагов.

## Статистический анализ

Размер выборки предварительно не рассчитывался ввиду редкости заболевания. Для представления количественных данных использована медиана (Me) с указанием интерквартильного диапазона [ Q25;Q75], максимальных и минимальных значений, качественные переменные представлены в виде абсолютных и относительных частот. Сравнение двух независимых групп для количественных данных выполнялось с помощью критерия Стьюдента для признаков, соответствующих закону нормального распределения, и критерия Манна–Уитни — для признаков, не соответствующих закону нормального распределения. Качественные переменные сравнивались между собой с помощью критерия Хи квадрат (χ2) и точного двустороннего критерия Фишера.

С целью выявления предикторов использовался метод логистической регрессии с определением достоверной значимости (р), отношения шансов (Odds Ratio, OR) и доверительных интервалов (95% ДИ). Проводился однофакторный имногофакторный регрессионный анализ прогностических факторов выживаемости с применением регрессионной модели пропорционального риска Кокса для нахождения независимых влияний ряда потенциальных предикторов на выживаемость пациентов. Переменные включались в модель, если p<0,050.

Для выявления прогностических пороговых значений отдельных предикторов использовался ROC-анализ. Анализировались следующие параметры: площадь под ROC-кривой (AUС), диагностическая чувствительность и диагностическая специфичность. При интерпретации показателя AUC использовали общепризнанную экспертную шкалу. Отрезная точка выбиралась с помощью критерия Юдена (разность суммы диагностической чувствительности и диагностической специфичности и единицы должна была быть максимальной для данной точки). Критический уровень статистической значимости для всех проверяемых гипотез был принят равным 0,050 (p<0,050).

Анализ выживаемости проводили по методу Каплана–Майера, кривые выживаемости сравнивали при помощи лог-рангового критерия.

Статистическая обработка данных осуществлялась при помощи пакета статистических программ IBM SPSS Statistics 23 (SPSS. Inc, Chicago, IL, USA).

## Этическая экспертиза

Протокол исследования одобрен локальным этическим комитетом ФГБУ «НМИЦ эндокринологии» Минздрава России, выписка из протокола №12 от 29.06.2022 г.

## РЕЗУЛЬТАТЫ

## Объекты (участники) исследования

В исследование включен 151 пациент (92 женщины, 59 мужчин). Медиана периода наблюдения пациентов — 50 мес [ 13; 91] с максимальным сроком наблюдения 382 мес. Возраст больных на момент диагностики заболевания составил от 12 до 76 лет (Ме 40 лет [ 28; 54]). Длительность заболевания с момента появления симптомов до верификации диагноза варьировала от 2 до 180 мес (Ме 17 мес [ 7; 44]), в среднем — 32,2 мес. Циклическое течение эндогенного гиперкортицизма, установленноена основании анализа анамнестических данных, а также подтвержденное результатами лабораторных исследований, зафиксировано в 11 случаях (7,3%). Общая характеристика пациентов с различной локализацией АКТГ-продуцирующих НЭО сведена в табл. 1. Локализация источника эктопической продукции АКТГ представлена на рис. 1. Длительность периода наблюдения пациентов с неустановленным первичным опухолевым очагом составила от 1 до 134 мес (Ме 18 мес [ 8; 54]).

**Table:** Таблица 1. Общая характеристика пациентов с АКТГ-продуцирующими НЭО различной локализацииTable 1. General characteristics of patients with ACTH-producing NETs of different localization Примечание: НЭО — нейроэндокринная опухоль; МРЩЖ — медуллярный рак щитовидной железы.

Локализация НЭО	Число женщин/мужчин	Средний возраст установкидиагноза,лет	Длительность симптомов до установки диагноза, мес	Период наблюдения, мес Me [ Q1; Q3] (min; max)	Ki-67, % Me [ Q1; Q3] (min; max)	Метастазы	Рецидив	Летальный исход
Легкое	51/41	42±15	Ме 22 [ 8; 48] (2; 180)	Ме 62 [ 19,3; 103] (1; 382)	Ме 2,6 [ 1,2; 6,0] (0,5; 18,8)	12/92	16/69	15/92
Тимус	11/6	31±12,9	Ме 13 [ 5,5; 37,5] (3; 76)	Ме 54 [ 26,5; 67,5] (2; 118)	Ме 18 [ 15; 32,3] (3,8; 50)	13/17	5/7	10/17
Надпочечник	4/1	51,2±9,6	Ме 26 [ 13,5; 48] (9; 62)	(68; 127)	Ме 3 [ 3; 14] (3; 24)	–	–	–
Поджелудочная железа	8/0	41,8±12,0	Ме 14 [ 5; 55,5] (4; 60)	Ме 8 [ 5,3; 38,8] (2; 162)	(1;6)	3/8	–	4/8
Слепая кишка	1/0	53	12	16	0,0	1/1	–	1/1
Червеобразный отросток	1/0	20	48	165	18,7	1/1	–	–
МРЩЖ	1/0	40	22	12	22,0	1/1	–	1/1

**Figure:**
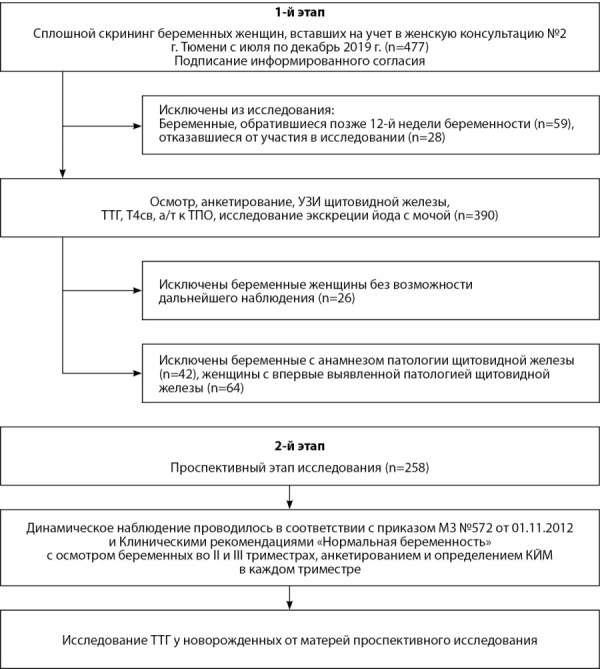
Рисунок 1. Локализация источника эктопической продукции АКТГ.Figure 1. Localization of the source of ectopic ACTH production.

Среди осложнений в активной стадии заболевания превалировали артериальная гипертензия (n=127 (84,1%)), сахарный диабет (n=84 (55,6%)), остеопороз (n=89 (58,9%)) с развитием низкотравматичных переломов в 66 случаях, заболевания сердечно-сосудистой системы (n=79 (52,3%)), включавшие стероидную кардиомиопатию, различные нарушения ритма и проводимости, хроническую сердечную недостаточность.

## Лечение пациентов с АКТГ-эктопированным синдромом и отдаленные результаты наблюдения

На момент исследования первичный очаг эктопической продукции АКТГ удален у 101 больного (66,9%). Радикальное хирургическое лечение не удалось провести 9 пациентам, среди них 4 имели НЭО поджелудочной железы с опухолевой инвазией крупных сосудов, магистральных венозных стволов и множественными метастазами в печени, 3 пациента — с НЭО тимуса и метастатическим поражением костей скелета, легких, лимфатических узлов, канцероматозом брюшины, у одной пациентки также верифицирован метастаз НЭО тимуса в яичнике, еще в 2 случаях НЭО бронхолегочной локализации радикальное хирургическое удаление невозможно вследствие особенностей расположения, характера роста объемного образования и высокого риска интраоперационных осложнений. Одна пациентка с НЭО легкого отказалась от хирургического вмешательства и умерла через 24 мес от осложнений новой коронавирусной инфекции в возрасте 72 лет.

Двусторонняя адреналэктомия по жизненным показаниям выполнена 42 пациентам (27,8%), из них 15 больных до установления локализации АКТГ-продуцирующей НЭО первоначально были подвергнуты операции в связи с тяжелым течением эндогенного гиперкортицизма и 15 пациентов — с неустановленной локализацией первичного опухолевого очага, остальным 12 пациентам с выраженными проявлениями гиперкортицизма удаление обоих надпочечников проведено в период рецидива заболевания после хирургического лечения первичного опухолевого очага и неудовлетворительных результатов длительной медикаментозной терапии и/или прогрессирования, несмотря на проводимую химиотерапию и/или лучевую терапию.

Для контроля симптомов эндогенного гиперкортицизма 55 пациентов (36,4%) получали медикаментозную терапию, из них 38 больных — аналоги соматостатина пролонгированного действия (октреотид 20–40 мг (n=28), ланреотид 120 мг (n=10)), в 24 случаях назначались ингибиторы стероидогенеза (кетоконазол 400–800 мг/сут (n=23), 1 больной получал мифепристон 300 мг/сут). Семи пациентам проводилась комбинированная терапия вышеперечисленными препаратами из различных фармакологических групп. Длительность приема медикаментозной терапии составила от 2 до 111 мес (Ме 18,5 мес [ 10,8; 32,5]) среди пациентов, получавших аналоги соматостатина, и от 2 до 48 мес (Ме 11,5 мес [ 3,8; 26]) в подгруппе пациентов, получавших блокаторы стероидогенеза. В структуре больных, которым назначалась медикаментозная терапия, 35 — с НЭО бронхолегочной локализации, 7 — с НЭО тимуса, 2 — с локализацией НЭО в поджелудочной железе, 1 — в надпочечнике, 10 — сНЭО неустановленной локализации. 14 больным потребовалось назначение химиотерапии, еще 5 пациентам проведена адъювантная лучевая терапия.

Метастатическое поражение регионарных лимфатических узлов и отдаленные метастазы в кости, печень, поджелудочную железу, почки, яичник, легкие, распространяющиеся на плевру, брюшину, выявлены в 23,2% случаев (35 пациентов).

Рецидив заболевания после удаления АКТГ-продуцирующей НЭО наблюдался в 24,4% (n=21/86) в сроки от 155 до 2653 дней (Ме 899 дней [ 279; 1442]), у 15 больных никогда не регистрировалась ремиссия заболевания, что в подавляющем большинстве случаев (n=10/15) было обусловлено наличием вторичных опухолевых очагов. По результатам проведенного исследования, наиболее часто рецидивировали НЭО тимуса — в 71,4% случаев (5/7). Рецидивы НЭО бронхолегочной локализации наблюдались значительно реже — в 23,2% (16/60). Случаев рецидива НЭО других локализаций не наблюдалось. Долгосрочная ремиссия сохранилась у 64 пациентов, что составило 74,4%. Длительность ремиссии в среднем была равной 67,6 мес (2097 дней), с Ме 53,5 мес [ 24; 105,3]), максимально 318 мес (Ме 1685,5 дня [ 744; 3268,8]). В послеоперационном периоде нам не удалось получить информацию об 1 пациенте, в связи с чем мы не располагаем данными о радикальности проведенного хирургического лечения.

Летальные исходы наступили у 42 пациентов (28%) в возрасте от 27 до 81 года, среди них 21 больной (50%) умер от полиорганной недостаточности вследствие прогрессирования заболевания, 6 пациентов — вследствие тромбоэмболии легочной артерии, в 5 случаях причиной смерти стали сердечно-сосудистые события (2 пациента умерли на 9-е и 10-е сутки после оперативного вмешательства вследствие острого нарушения мозгового кровообращения с развитием отека, дислокации ивклинения головного мозга в большое затылочное отверстие, у 2 пациентов констатирована внезапная сердечная смерть, 1 больная умерла вследствие обширного инфаркта миокарда), в 2 случаях смерть наступила в результате массивного кровотечения в послеоперационном периоде, в 1 случае причиной летального исхода стал синдром диссеминированного внутрисосудистого свертывания, развившийся на 10-е сутки после двусторонней адреналэктомии, 3 пациента умерли от осложнений новой коронавирусной инфекции (COVID-19), у 1 больной летальный исход наступил через 6 мес после перелома шейки бедра в возрасте 81 года, причина смерти 3 пациентов неизвестна. В 10 случаях первичный опухолевый очаг оставался неустановленным даже после проведения аутопсии. 26 пациентов умерли в активной стадии заболевания, из них у 6 отсутствовала ремиссия после удаления первичного опухолевого очага, так как имелись регионарные и отдаленные метастазы.

## Факторы, ассоциированные с развитием неблагоприятного исхода

За период наблюдения, максимально составивший 31 год и 8 мес, летальный исход наступил у 42 пациентов, 1 пациент выбыл из наблюдения. Таким образом, оценка предикторов выживаемости и летальных исходов проводилась на выборке из 150 пациентов. Средний возраст выживших составил 47,0±15,2 против 53,5±15,6 года у умерших (р=0,022). Время до наступления летального исхода с момента постановки диагноза составило в среднем 32 мес, Ме 16,5 мес [ 7; 54]. Влияния пола на выживаемость не выявлено: скончались 19% мужчин, включенных в исследование, и 34% женщин (p=0,062). Описательная статистика и результаты сравнения групп выживших и умерших пациентов по изученным параметрам, включенным в анализ предикторов выживаемости, представлены в табл. 2.

**Table:** Таблица 2. Исследованные параметры в группах выживших и умерших пациентов с АКТГ-эктопированным синдромомTable 2. Parameters studied in groups of surviving and deceased patients with ACTH-ectopic syndrome Примечание: * — показатели в обеих группах статистически значимо различались.

Параметр	Выжившие	Умершие	р
Пол мужской	47 (43,5%)	11 (26,2%)	0,062
Пол женский	61 (56,5%)	31 (73,8%)
Возраст установки диагноза, лет	39 [ 26; 53]	52,5 [ 37,5; 60,3]	0,001*
Длительность заболевания до момента установки диагноза	22 [ 8; 47,5]	12,5 [ 6; 39,5]	0,075
Индекс массы тела, кг/м2	27,6 [ 23,9; 31,8]	27,6 [ 24; 31,1]	0,870
Двусторонняя адреналэктомия
Выполнялась	27 (25%)	14 (33,3%)	0,314
Не выполнялась	81 (75%)	28 (66,7%)
Локализация НЭО
НЭО легкого	76 (83,5%)	15 (16,5%)	0,001*
НЭО тимуса	7 (41,2%)	10 (58,8%)	0,007*
НЭО надпочечника (феохромоцитома)	5 (100%)	0	0,322
НЭО поджелудочной железы	4 (50%)	4 (50%)	0,220
НЭО слепой кишки	0	1 (100%)	1,000
НЭО червеобразного отростка	1 (100%)	0	1,000
Медуллярный рак щитовидной железы	0	1 (100%)	1,000
Неустановленная локализация	15	11	0,093
Индекс Ki-67, %	3,0 [ 1,5; 8,1]	11,0 [ 2,0; 21]	0,024*
Регионарные и отдаленные метастазы
Есть метастазы	13 (12%)	22 (52,4%)	<0,001*
Нет метастазов	95 (88%)	20 (47,6%)
Осложнения в активной стадии заболевания
I. Артериальная гипертензия
Есть артериальная гипертензия	89 (82,4%)	37 (88,1%)	0,466
Нет артериальной гипертензии	19 (17,6%)	5 (11,9%)
II. Заболевания сердечно-сосудистой системы
Имеются	49 (45,4%)	29 (69%)	0,011*
Отсутствуют	59 (54,6%)	13 (31%)
III. Сахарный диабет
Есть сахарный диабет	55 (50,9%)	29 (69%)	0,066
Нет сахарного диабета	53 (49,1%)	13 (31%)
IV. Остеопороз
Нормальная минеральная плотность кости или остеопения, переломов нет	46 (42,6%)	16 (38,1%)	0,572
Остеопороз без переломов	13 (12%)	10 (23,8%)
Остеопороз с множественными низкотравматичными переломами	49 (45,4%)	16 (38,1%)
Лабораторные обследования на момент установки диагноза
АКТГ в 8:00, пг/мл	135,5 [ 101,5; 187,4]	190 [ 136; 275,3]	0,001*
АКТГ в 23:00, пг/мл	115,4 [ 87,0; 167,8]	148,1 [ 107,5; 207,7]	0,010*
Кортизол в слюне в 23:00, нмоль/л	70,1 [ 42,3; 111,5]	161,2 [ 95,0; 434,6]	<0,001*
Кортизол в суточной моче, нмоль/сут	2780 [ 1738; 5089]	4670 [ 2635; 7749,1]	0,007*
Кортизол в крови в 23:00, нмоль/л	1196 [ 912,5; 1425,8]	1165,5 [ 1049,1; 1438,3]	0,431

Как видно из таблицы, пациенты двух групп сравнения достоверно различались по многим клиническим и лабораторным параметрам, а также по локализации АКТГ-секретирующей НЭО. Так, например, возраст установки диагноза АКТГ-эктопированного синдрома в подгруппе умерших больных оказался значительно больше (р=0,001), у лиц с летальным исходом также чаще регистрировались заболевания сердечно-сосудистой системы (р=0,011), однако частота других осложнений эндогенного гиперкортицизма статистически значимо не различалась в подгруппах выживших и умерших. В подгруппе пациентов, у которых наступил летальный исход, выявлены более высокие значения АКТГ и кортизола. Кроме того, регионарные и отдаленные метастазы статистически значимо чаще отмечались в подгруппе умерших больных (р<0,001), так же, как и индекс Ki-67 в удаленных опухолевых тканях был выше у данной категории пациентов (р=0,024).

Результаты ROC-анализа с определением площади под кривой операционных характеристик (AUC — Area Under Curve) для возраста диагностики заболевания и гормональных показателей как для предикторов летального исхода, а также соответствующие значения чувствительности и специфичности для оптимальных точек разделения (cut-off) представлены в табл. 3. По результатам ROC-анализа наибольшей прогностической ценностью обладала концентрация свободного кортизола вслюне, собранной в 23:00 ч, на момент верификации эндогенного гиперкортицизма вследствие АКТГ-эктопированного синдрома до хирургического или медикаментозного вмешательства: AUC составила 0,783; 95% ДИ 0,696–0,869, что свидетельствует о хорошей диагностической точности. При оценке диагностических параметров было определено, что оптимальное пороговое значение, то есть точка cut-off для кортизола в вечерней слюне, составляет 122,2 нмоль/л. Превышение показателя более 122,2 нмоль/л предсказывает риск летального исхода с чувствительностью 67,6% и специфичностью 81,1%.

**Table:** Таблица 3. Результаты ROC-анализа достоверных предикторов развития летального исходаTable 3. Results of ROC analysis of significant predictors of lethal outcome Примечание: ДЧ — диагностическая чувствительность; ДС — диагностическая специфичность.

Параметр, точка разделения (cut-off)	ДЧ, %	ДС, %	Площадь под кривой (AUC)	Стандартная ошибка	Асимптоматическая значимость	Асимптоматический 95% доверительный интервал
Нижняя граница	Верхняя граница
Возраст ≥51 года	54,8	73,1	0,673	0,048	0,001	0,578	0,767
АКТГ 8:00 ≥145,2 пг/мл	73,8	61,3	0,683	0,049	0,001	0,587	0,779
АКТГ 23:00 ≥128,1 пг/мл	64,3	59,4	0,636	0,049	0,010	0,540	0,733
Кортизол в слюне в 23:00 ≥122,2 нмоль/л	67,6	81,1	0,783	0,044	0,000	0,696	0,869
Кортизол в суточной моче ≥4564 нмоль/сут	53,7	69,9	0,644	0,052	0,007	0,542	0,745

Анализ ROC-кривых для остальных показателей продемонстрировал низкую диагностическую ценность, что не позволяет рассматривать данные параметры в качестве высокоточных предикторов неблагоприятного исхода (табл. 3). AUC для индекса Ki-67 по результатам ROC-анализа составила 0,664 (95% ДИ 0,500–0,828), однако пересекает диагональную опорную линию, демонстрируя низкую диагностическую ценность данного параметра, что может быть обусловлено недостаточным количеством наблюдений и большим разбросом значений для опухолей различных локализаций. Возможно, при наборе большей исследуемой группы индекс Ki-67 мог показать независимое прогностическое значение в нашей когорте пациентов.

Таким образом, в результате пошагового логистического регрессионного анализа отобрана модель с независимыми предикторами, повышающими риск наступления летального исхода, уровень значимости которых не достигал 0,050, включившая 9 факторов, из которых 4 сохранили статистическую значимость в составе многофакторной регрессионной модели Кокса пропорционального риска наступления неблагоприятного исхода. Итоговые результаты многофакторной регрессионной модели Кокса представлены в табл. 4. Исходя из анализа, значимое влияние на выживаемость больных с АКТГ-эктопированным синдромом оказывают возраст установки диагноза, в частности возраст ≥51 года является неблагоприятными фактором (OR 4,493; 95% ДИ 2,056–9,818; р<0,001); локализация НЭО, причем НЭО бронхолегочной локализации, наоборот, является протективным фактором (OR 0,281; 95% ДИ 0,119–0,665; р=0,004); в то время как наличие метастазов (OR 2,489; 95% ДИ 1,141–5,427; р=0,022), а также концентрация свободного кортизола в слюне, собранной в 23:00 ч, ≥122,2 нмоль/л (OR 2,493; 95% ДИ 1,014–6,128; р=0,047) (рис. 2) являются неблагоприятными факторами.

**Table:** Таблица 4. Многофакторный регрессионный анализ Кокса предикторов летального исходаTable 4. Multivariate Cox regression analysis of predictors of death Примечание: B — коэффициент в регрессии Кокса; SE — стандартная ошибка для коэффициента регрессии Кокса; Wald — χ2 Вальда проверяет нулевую гипотезу о том, что относительный риск летального исхода, связанный с данной переменной, равен единице; значимость — достигнутый уровень значимости для критерия χ2 Вальда; Exp(B) — отношение шансов, представляет собой повышенный или пониженный риск достижения конечной точки (летальный исход) в любой момент времени, связанный с единичным увеличением соответствующего ему параметра, с учетом эффекта всех остальных предикторов; Exp(B)>1 означает повышенный шанс наступления неблагоприятного исхода (то есть фактор имеет прямую связь с наступлением летального исхода); Exp(B)<1 — пониженный шанс достижения конечной точки в ходе исследования (то есть фактор является протективным и имеет обратную связь с вероятностью наступления неблагоприятного исхода); НЭО — нейроэндокринная опухоль.

Переменные в уравнении
Фактор	B	SE	Wald	статистическая связь	Значимость	Exp(B)	ДИ 95,0% для Exp(B)
нижняя	верхняя
Возраст на момент установки диагноза ≥51 года	1,502	0,399	14,192	1	0,000	4,493	2,056	9,818
НЭО бронхолегочной локализации	-1,269	0,440	8,336	1	0,004	0,281	0,119	0,665
НЭО тимуса	0,556	0,541	1,058	1	0,304	1,120	0,087	14,427
Наличие метастазов	0,912	0,398	5,254	1	0,022	2,489	1,141	5,427
АКТГ 8:00 ≥145,2 пг/мл	0,080	0,720	0,012	1	0,911	1,084	0,264	4,446
АКТГ 23:00 ≥128,1 пг/мл	0,939	0,583	2,596	1	0,107	2,557	0,816	8,012
Кортизол в слюне в 23:00 ≥122,2 нмоль/л	0,913	0,459	3,962	1	0,047	2,493	1,014	6,128
Кортизол в суточной моче ≥4564 нмоль/сут	0,736	0,678	1,180	1	0,277	2,088	0,553	7,882
Сердечно-сосудистые заболевания в активной стадии гиперкортицизма	0,363	0,363	1,002	1	0,317	1,438	0,706	2,930

**Figure:**
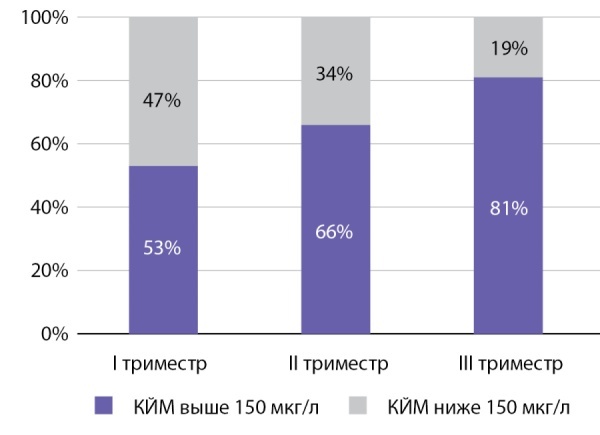
Рисунок 2. Общая выживаемость пациентов в зависимости от концентрации кортизола в вечерней слюне.Figure 2. Overall survival of patients depending on the concentration of cortisol in the evening saliva.

Общая выживаемость пациентов с АКТГ-эктопированным синдромом в зависимости от локализации НЭО представлена на рис. 3. Существующие различия между группами по изучаемому показателю признаны статистически значимыми, pLog-Rank <0,001. Пациенты с бронхолегочными карциноидами имели достоверно лучшую выживаемость по сравнению с НЭО других локализаций (р=0,006). Выживаемость больных с оккультными НЭО была сопоставимой с выживаемостью больных с локализацией НЭО в поджелудочной железе (р=0,387) и не отличалась от выживаемости пациентов с НЭО тимуса (р=0,755).

**Figure:**
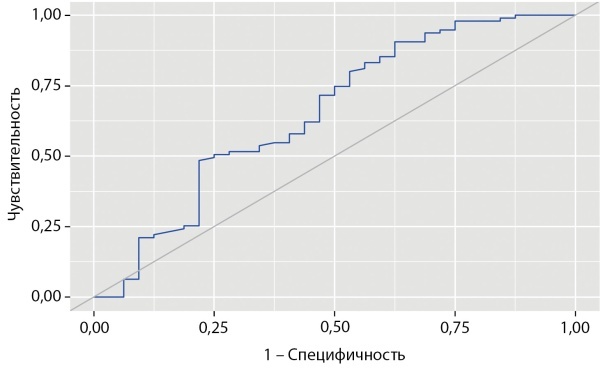
Рисунок 3. Общая выживаемость пациентов с АКТГ-эктопированным синдромом в зависимости от локализации НЭО (кривые выживаемости Каплана–Майера).Figure 3. Overall survival of patients with ACTH-ectopic syndrome depending on the location of the NET (Kaplan-Meier survival curves).

При анализе полученных показателей выживаемости выявлено, что 3- и 5-летняя выживаемость пациентов с локализацией НЭО в легком составила 93 и 86% соответственно, для НЭО тимуса эти показатели были 82 и 60% соответственно. Достоверно худшие показатели 3- и 5-летней выживаемости имели пациенты с неустановленным источником АКТГ-эктопированного синдрома — 56 и 50% соответственно. Ввиду того, что медиана периода наблюдения пациентов с НЭО поджелудочной железы составила 8 мес, а средний период наблюдения — 31 мес, показатели общей выживаемости для данной подгруппы больных рассчитаны не были.

Медиана выживаемости пациентов с отдаленными метастазами составила 56 мес [ 41,3; 66,7]. Показатели общей 3- и 5-летней выживаемости больных без метастазов составили 85 и 83% против 70 и 50% соответственно при наличии вторичных опухолевых очагов. Выраженная тенденция к более низким показателям выживаемости наблюдалась в подгруппе пациентов с НЭО тимуса и наличием отдаленных метастазов: 3- и 5-летняя выживаемость составила 77 и 59% против 90 и 70% соответственно для пациентов с бронхолегочными карциноидами (рис. 4).

**Figure:**
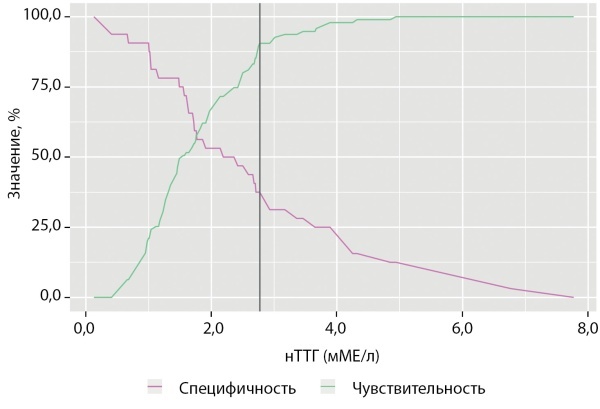
Рисунок 4. Общая выживаемость пациентов в зависимости от распространенности опухолевого процесса.Figure 4. Overall survival of patients depending on the prevalence of the tumor process.

Мы также провели оценку влияния двусторонней адреналэктомии на выживаемость больных. Двусторонняя адреналэктомия, как правило, проводилась наиболее тяжелым пациентам по жизненным показаниям. В нашей когорте двусторонняя адреналэктомия проводилась у впервые выявленных пациентов в связи с тяжелым течением гиперкортицизма НЭО (n=15), в том числе при неустановленной локализации новообразования (n=15) или в период рецидива заболевания (n=12). При сравнении пациентов, перенесших двустороннюю адреналэктомию из-за более тяжелого течения гиперкортицизма, c теми, кому такое лечение не выполнялось (n=109), не было выявлено статистически значимых различий по показателям выживаемости, как и не получено достоверных различий при сравнении одновременно между всеми группами (pLog-Rank=0,401).

## ОБСУЖДЕНИЕ

Данная работа представляет собой первое крупное исследование в России, посвященное анализу предикторов выживаемости пациентов с АКТГ-эктопированным синдромом. Главным результатом настоящего исследования явился комплексный анализ широкого спектра факторов, позволивший выделить из множества потенциальных предикторов наиболее влиятельные, независимо ассоциированные с выживаемостью пациентов в отдаленном периоде наблюдения.

Установлено, что более молодой возраст на момент диагностики заболевания является независимым протективным фактором выживаемости. В нашем исследовании пороговое значение возраста установки диагноза, достоверно повышающее риск неблагоприятного исхода, составило ≥51 года. Подобные результаты были получены в исследовании Davi M.B. и соавт. [9], включившем 110 больных с АКТГ-эктопированным, синдромом. Пациенты в возрасте ≥60 лет на момент диагностики заболевания имели достоверно худший прогноз в сравнении с более молодыми пациентами. Общая 5-летняя выживаемость в данной подгруппе составила 52% против 81% для больных, возраст которых на момент установки диагноза не превышал 40 лет, и 77% для тех, чей возраст находился в диапазоне от 40 до 59 лет (р=0,016).

В ходе настоящего исследования высокую прогностическую значимость в определении риска летального исхода продемонстрировала концентрация свободного кортизола в слюне в вечернее время суток. Стоит отметить, что в однофакторных регрессионных моделях другие гормональные показатели, стандартно оцениваемые у пациентов с эндогенным гиперкортицизмом, также достоверно ассоциировались с выживаемостью больных, однако только кортизол слюны сохранил статистическое значение в многофакторной модели. Превышение отрезной точки в 122,2 нмоль/л, что в 12,7 раза выше значения верхней границы референсного интервала, ассоциировано с высоким риском развития летального исхода.

Аналогично нашему исследованию в работе Davi M.B. и соавт. [9] ряд факторов, показавших ассоциацию с выживаемостью больных с АКТГ-продуцирующими НЭО, был сопоставим с набором факторов, полученных нами в однофакторном анализе, предшествовавшем построению многофакторной модели. Так, например, в вышеупомянутом исследовании худшую выживаемость имели пациенты с более чем 5-кратным увеличением концентрации свободного кортизола в суточной моче (p=0,005), а также больные с >5-кратным превышением верхней границы референсного интервала для АКТГ (р=0,001). Стоит отметить, что первый фактор сохранил свою прогностическую значимость в многофакторной модели. Кроме того, в однофакторной модели вероятность летального исхода оказалась выше в подгруппе с >3-кратным увеличением кортизола в утренние часы (р<0,001). Аналогичные результаты были получены в другом зарубежном исследовании, хотя влияние данного фактора отмечено только при однофакторном, но не многофакторном анализе [10]. В нашей работе мы не проводили измерение кортизола в крови в ранние утренние часы ввиду низкой диагностической информативности данного теста, следовательно, прогностическая значимость данного показателя нами не оценивалась.

Выраженный гиперкортицизм в подавляющем большинстве случаев сопровождается развитием широкого спектра осложнений, что значительно ухудшает прогноз и приводит к стойкой инвалидизации. В нашей работе при проведении однофакторного регрессионного анализа было установлено, что факт наличия у пациента сердечно-сосудистого осложнения увеличивал риск неблагоприятного исхода, однако другие осложнения заболевания статистически значимо не различались между группами. Напротив, в исследовании Lase I. и соавт. [10] отношение шансов летального исхода было выше при наличии сахарного диабета, однако в многофакторном анализе данный фактор не оказывал достоверного влияния на выживаемость. В другой работе, Davi M.B. и соавт. [9], единственным отрицательным прогностическим фактором было также развитие у пациента сахарного диабета (р=0,0146). Факт наличия других сопутствующих заболеваний не показал достоверной связи с летальным исходом. В этом же исследовании высокую значимость в определении прогноза продемонстрировало наличие вторичных опухолевых очагов. Пациенты с метастазами имели достоверно худший прогноз, что согласуется с результатами, полученными в нашей работе. Показатели 5-летней выживаемости при прогрессировании опухолевого процесса и наличии вторичных опухолевых очагов в нашем исследовании были аналогичны результатам зарубежных исследователей, где показатель 5-летней выживаемости составляет от 47 до 60% [9][11][12].

Согласно данным литературы, НЭО бронхолегочной локализации являются наиболее частой причиной АКТГ-эктопированного синдрома [13][14], что подтверждают результаты проведенного нами исследования, в котором опухоли данной локализации были выявлены в 60,9% случаев. Хирургическое лечение НЭО, являющейся источником эктопической продукции АКТГ, является основным методом лечения АКТГ-эктопированного синдрома [15]. Как показало наше исследование, радикальное удаление первичного опухолевого очага позволяет добиться ремиссии заболевания в 74,4% случаев, что значительно выше, чем сообщалось в литературе. Наилучшим послеоперационным прогнозом обладают высокодифференцированные НЭО бронхолегочной локализации, частота развития рецидивов не превышает 25%. Кроме того, пациенты с бронхолегочными карциноидами имели достоверно лучшую выживаемость по сравнению с НЭО других локализаций, что также подтверждается в работах зарубежных исследователей [3][9][12]. Примечательно, что выживаемость пациентов с НЭО неустановленной локализации и НЭО поджелудочной железы в нашем исследовании была сопоставимой в отличие от результатов, полученных Ilias I. и соавт. [3], где больные с оккультными НЭО, напротив, имели лучшие показатели выживаемости, практически сопоставимые с выживаемостью пациентов с бронхолегочными карциноидами. В нашем исследовании исход для НЭО поджелудочной железы, так же, как и для НЭО тимуса, был значительно хуже по сравнению с бронхолегочными карциноидами, что выражалось в более низкой частоте хирургического лечения, низких показателях выживаемости и большей частоте прогрессирования заболевания сразвитием отдаленных метастазов.

Вопрос о выполнении двусторонней адреналэктомии у пациентов с АКТГ-эктопированным синдромом при наличии выраженного гиперкортицизма, связанных с ним осложнений, метастазов или в случае невозможности обнаружения первичного опухолевого очага остается особенно актуальным. Почти 30% пациентов в нашей когорте подверглись данной операции, из них в 71,4% случаев двусторонняя адреналэктомия проводилась в качестве первого метода лечения, и только в половине случаев впоследствии удалось установить локализацию НЭО. В опубликованном ранее исследовании пациенты с АКТГ-эктопированным синдромом, перенесшие двустороннюю адреналэктомию, имели лучшую выживаемость по крайней мере в первые 2 года, что подчеркивает важность быстрого контроля гиперкортицизма. Несмотря на то что мы не выявили достоверных различий по выживаемости, двусторонняя адреналэктомия является единственным эффективным методом, позволяющим добиться быстрого наступления терапевтического эффекта, избавив пациента от всего спектра проявлений и осложнений гиперкортицизма.

## Клиническая значимость результатов

На основе анализа большого клинического материала и метода логистической регрессии, направленного на пошаговый отбор наиболее значимых предикторов с проведением оценки прогностической ценности получаемого в модели результата, определена совокупность наиболее значимых предикторов летальности пациентов с АКТГ-эктопированным синдромом, а также выявлены диагностические пороговые уровни для признаков, имеющих количественное выражение, что определяет достоверность, уникальность и значимость результатов исследования. Полученные данные позволяют выделить группу высокого риска, требующую более тщательного наблюдения, оптимизации подходов к диагностике и выбору оптимальных методов лечения.

## Ограничения исследования

С ретроспективным дизайном связано значительное количество пропусков в данных, не позволившее включить в многомерный анализ, в частности, такой перспективный предиктор, как индекс пролиферативной активности Ki-67. По ряду ограничительных причин полученные в ходе исследования кривые выживаемости для индивидуального прогноза продолжительности жизни должны быть использованы с осторожностью.

## ЗАКЛЮЧЕНИЕ

Предикторами выживаемости пациентов с АКТГ-эктопированным синдромом являются возраст установки диагноза, локализация НЭО, метастазы и концентрация свободного кортизола в вечерней слюне исходно на момент установки диагноза дохирургического или медикаментозного вмешательства. Диагностика и профилактика осложнений заболевания и жизнеугрожающих состояний у пациентов с выраженным гиперкортицизмом должна проводиться безотлагательно во избежание летального исхода. Учитывая, что не всегда назначение комбинированных схем медикаментозной терапии позволяет достичь контроля и ремиссии гиперкортицизма, оптимальным решением в ряде случаев является выполнение двусторонней адреналэктомии.

## ДОПОЛНИТЕЛЬНАЯ ИНФОРМАЦИЯ

Источники финансирования. Исследование проведено при поддержке Российского научного фонда (грант РНФ 19-15-00398-П).

Конфликт интересов. Авторы декларируют отсутствие явных и потенциальных конфликтов интересов, связанных с содержанием настоящей статьи.

Участие авторов. Голоунина О.О. — сбор и обработка материала, формирование электронной базы данных, статистическая обработка, анализ полученных результатов, написание основного текста рукописи; Белая Ж.Е., Рожинская Л.Я. — концепция и дизайн исследования, научное руководство проводимым исследованием, ведение пациентов, редактирование текста рукописи; Пикунов М.Ю. — хирургическое лечение пациентов с нейроэндокринными опухолями бронхолегочной локализации, редактирование текста рукописи; Кузнецов Н.С. — выполнение двусторонней адреналэктомии, редактирование текста рукописи; Маркович А.А., Дзеранова Л.К., Марова Е.И., Фадеев В.В. — ведение пациентов, редактирование текста рукописи; Мельниченко Г.А., Дедов И.И. — редактирование текста, одобрение финальной версии рукописи. Все авторы одобрили финальную версию статьи перед публикацией, выразили согласие нести ответственность за все аспекты работы, подразумевающую надлежащее изучение и решение вопросов, связанных с точностью или добросовестностью любой части работы.

## References

[cit1] Lacroix A, Feelders RA, Stratakis CA, Nieman LK. Cushing’s syndrome. Lancet. 2015;386(9996):913-927. doi: https://doi.org/10.1016/S0140-6736(14)61375-1 26004339

[cit2] Feelders Richard, Sharma Susmeeta, Nieman Lynnette (2015). Cushing's syndrome: epidemiology and developments in disease management. Clinical Epidemiology.

[cit3] Ilias Ioannis, Torpy David J., Pacak Karel, Mullen Nancy, Wesley Robert A., Nieman Lynnette K. (2005). Cushing’s Syndrome Due to Ectopic Corticotropin Secretion: Twenty Years’ Experience at the National Institutes of Health. The Journal of Clinical Endocrinology & Metabolism.

[cit4] Golounina O.O., Belaya Zh.E., Rozhinskaya L.Ya., i dr. Kliniko-laboratornaya kharakteristika i rezul'taty lecheniya patsientov s AKTG-produtsiruyushchimi neiroendokrinnymi opukholyami razlichnoi lokalizatsii // Terapevticheskii arkhiv. — 2021. — T. 93. — №10. — S. 1171-1178. 36286818 10.26442/00403660.2021.10.201102

[cit5] Mel'nichenko G.A., Dedov I.I, Belaya Zh.E., i dr. Bolezn' Itsenko–Kushinga: klinika, diagnostika, differentsial'naya diagnostika, metody lecheniya // Problemy endokrinologii. — 2015. — T. 61. — №2. — S. 55-77.

[cit6] Belaya Zh.E., Rozhinskaya L.Ya., Mel'nichenko G.A., i dr. Rol' gradienta prolaktina i AKTG/prolaktin-normalizovannogo otnosheniya dlya povysheniya chuvstvitel'nosti i spetsifichnosti selektivnogo zabora krovi iz nizhnikh kamenistykh sinusov dlya differentsial'noi diagnostiki AKTG-zavisimogo giperkortitsizma // Problemy endokrinologii. — 2013. — T. 59. — №4. — S. 3-10.

[cit7] Dedov I.I., Belaya Zh.E., Sitkin I.I., i dr. Znachenie metoda selektivnogo zabora krovi iz nizhnikh kamenistykh sinusov v differentsial'noi diagnostike AKTG- zavisimogo endogennogo giperkortitsizma // Problemy endokrinologii. — 2009. — T. 55. — №6. — S. 35-40.

[cit8] Rindi G, Mete O, Uccella S, et al. Overview of the 2022 WHO Classification of Neuroendocrine Neoplasms. Endocr Pathol. 2022;33(1):115-154. doi: https://doi.org/10.1007/s12022-022-09708-2 35294740

[cit9] Davi’ Maria Vittoria, Cosaro Elisa, Piacentini Serena, Reimondo Giuseppe, Albiger Nora, Arnaldi Giorgio, Faggiano Antongiulio, Mantovani Giovanna, Fazio Nicola, Piovesan Alessandro, Arvat Emanuela, Grimaldi Franco, Canu Letizia, Mannelli Massimo, Ambrogio Alberto Giacinto, Pecori Giraldi Francesca, Martini Chiara, Lania Andrea, Albertelli Manuela, Ferone Diego, Zatelli Maria Chiara, Campana Davide, Colao Annamaria, Scaroni Carla, Terzolo Massimo, De Marinis Laura, Cingarlini Sara, Micciolo Rocco, Francia Giuseppe (2017). Prognostic factors in ectopic Cushing’s syndrome due to neuroendocrine tumors: a multicenter study. European Journal of Endocrinology.

[cit10] Lase Ieva, Strele Ieva, Grönberg Malin, Kozlovacki Gordana, Welin Staffan, Janson Eva Tiensuu (2020). Multiple hormone secretion may indicate worse prognosis in patients with ectopic Cushing’s syndrome. Hormones.

[cit11] Kamp K, Alwani R A, Korpershoek E, Franssen G J H, de Herder W W, Feelders R A (2015). Prevalence and clinical features of the ectopic ACTH syndrome in patients with gastroenteropancreatic and thoracic neuroendocrine tumors. European Journal of Endocrinology.

[cit12] Isidori Andrea M., Kaltsas Gregory A., Pozza Carlotta, Frajese Vanni, Newell-Price John, Reznek Rodney H., Jenkins Paul J., Monson John P., Grossman Ashley B., Besser G. Michael (2006). The Ectopic Adrenocorticotropin Syndrome: Clinical Features, Diagnosis, Management, and Long-Term Follow-Up. The Journal of Clinical Endocrinology & Metabolism.

[cit13] Margaritora Stefano, Cardillo Giuseppe, Filosso Perluigi, Novellis Pierluigi, Rapicetta Cristian, Carleo Francesco, Bora Giulia, Cesario Alfredo, Stefani Alessandro, Rossi Giulio, Paci Massimiliano, Lococo Filippo (2015). Bronchopulmonary Carcinoids causing Cushing Syndrome: Results from a Multicentric Study Suggesting a More Aggressive Behavior. The Thoracic and Cardiovascular Surgeon.

[cit14] Sathyakumar Samantha, Paul Thomas Vizhalil, Asha Hesargatta Shyamsunder, Gnanamuthu Birla Roy, Paul M.J., Abraham Deepak Thomas, Rajaratnam Simon, Thomas Nihal (2017). Ectopic Cushing Syndrome: A 10-Year Experience from a Tertiary Care Center in Southern India. Endocrine Practice.

[cit15] Pikunov M.Yu., Pechetov A.A., Golounina O.O., i dr. Osobennosti podgotovki i khirurgicheskikh aspektov lecheniya patsientov s AKTG-produtsiruyushchimi neiroendokrinnymi opukholyami legkikh // Endokrinnaya khirurgiya. — 2021. — T. 15. — №2. — S. 4-12.

